# Couple-kissing flaps for successful repair of severe sacral pressure ulcers in frail elderly patients

**DOI:** 10.1186/s12877-017-0680-4

**Published:** 2017-12-11

**Authors:** Jing-Chun Zhao, Bo-Ru Zhang, Kai Shi, Jia-Ao Yu, Jian Wang, Qing-Hua Yu, Lei Hong

**Affiliations:** Burns and Plastic Reconstruction Unit, The First Hospital of Jilin University, No. 71 Xinmin Street, Changchun, 130021 China

**Keywords:** Pressure ulcer, Sacrum, Surgical flaps, Elderly patients

## Abstract

**Background:**

Surgical repair of severe pressure ulcers (PUs) in elderly patients remains a challenge for clinicians due to the complicated comorbidities and the special physical characteristics of elderly patients. The objective of this study was to evaluate the application of couple-kissing flaps (CKF) in the reconstruction of sacral PUs in these patients.

**Methods:**

Elderly patients (over 70 years) with stage 3 or stage 4 PUs who underwent CKF immediately after radical debridement between July 2012 and December 2015 were enrolled in this retrospective study. Patients’ demographics were extracted from the medical records.

**Results:**

A total of 12 patients were involved in this study. The average age of the patients was 76.83 years (ranged from 71 to 92 years). The donor site was closed primarily in all cases. All the flaps healed uneventfully without complications. Follow-up observations were conducted for an average of 13.6 months (ranged from 9 months to 2 years). Cosmetic results were satisfactory, with no surgical site breakdown or recurrence of PU in any of the cases. Three representative cases are presented.

**Conclusions:**

The CKF is a reliable and satisfactory option for the reconstruction of severe sacral PUs defects in elderly patients. CKF is associated with an relatively low rate of complications and recurrence.

## Background

Pressure ulcers (PUs)—also known as pressure injury, pressure sores, bedsores, or decubitus ulcers—are one of the most common skin injuries. According to the National Pressure Ulcer Advisory Panel (NPUAP) 2016, pressure ulcer is redefined as pressure injury: a localized damage to the skin and underlying soft tissue usually over a bony prominence or related to a medical or other device [1].

PUs are more common in individuals bedridden for extended periods in the supine position or using a wheelchair. In addition, malnutrition, arteriosclerosis, paralysis, or neuropathy are also believed to play important roles in the occurrence/recurrence of PUs due to poor blood perfusion to tissues or reduced sensation of skin [2].

The most common involved sites of PUs are the skin overlying the sacrum, coccyx, ischial tuberosity, or the trochanters, but other sites such as elbows, knee, iliac crest, and lateral malleolus can also be affected [3]. PUs can lead to patient suffering and frequent and lengthy hospitalizations that include intensive nursing interventions and financial burdens for the patient, family, and the healthcare system [4].

Based on the latest classification system of NPUAP 2016, PU can be divided into four different stages. Commonly, the healing process of minor to moderate PUs (NPUAP stage 1 and 2) may be slowed by ageing, medical conditions (such as diabetes or infection), smoking habits, or medications such as anti-inflammatory drugs. However, to date, the optimal treatment method for severe PUs (NPUAP stage 3, 4 and unstageable/deep tissue pressure injury) remains challenging in clinical settings and may typically require surgical intervention.

Pressure ulcers are a global problem with prevalence range from 3.4% to 10.9% in the hospitalized or emergency department setting [3, 5, 6]. For neurologically impaired patients or critically ill patients stayed in the intensive care unit, the incidence may be much higher—from 9% to 34.4% [7–10]. Pressure ulcers are a major health safety hazard as they increase the risk of expensive hospitalization and death [11]. The overall prevalence of stage 2–4 PUs at nursing home admission in the United States ranges between 5% and 20%, and the costs to treat severe PUs were found to be substantially higher and entail a substantial financial concern for all involved parties [11, 12]. The more severe the pressure ulcer, the more costly and longer time to heal.

As a major public healthcare issue affecting hospital and community patient populations, the prevention of occurrence/recurrence of severe PUs and early intervention needs more focus and improvement. Multidisciplinary care and treatment principles, combined with educational programs led by healthcare providers, are mandatory to achieve fewer PUs along with faster healing and less recurrence and complications.

Presently many technical modalities (e.g., negative pressure device [13], extracellular, collagen-rich matrix [14]); arginine-enriched oral nutritional supplementation [15]; and surgical techniques (e.g., muscular flap,cutaneous flaps, myocutaneous flap, perforator/fascia pedicle–based flap [16, 17]) have been described in the treatment of severe PUs defects. However, few studies have focused on the treatment of severe sacral PUs in geriatric populations.

As the elderly comprise over 70% of people affected with PUs, and with the expected increase in longevity in aging societies, the prevalence and incidence of PUs in this high-risk population will likely increase alarmingly [18, 19]. Moreover, the treatment of stage-4 PUs represents a major health burden and can be difficult and expensive to treat.

In this study, we describe a novel design of couple-kissing flap (CKF) that extends the range of therapeutic modalities in elderly patients with severe sacral PUs. It could provide advantages over traditional advanced flap procedures and result in satisfactory functional and aesthetic outcomes.

## Methods

### Ethical approval

This study was approved by the Institutional Review Board from the First Hospital of Jilin University; all participants or their legal guardians provided informed consent in person.

### Patients, inclusion and exclusion criteria

The CKF method was applied in elderly patients admitted to restore severe sacral PUs defects during the period of 2012–2015 in our unit. Patients were eligible for inclusion if they were: aged 70 years or above, suffered NPUAP stage 3, 4 or deep tissue pressure injury, not in need of life-saving support, and underwent CKF surgery immediately to close the resultant defects (larger than 7 cm midline width of defect after radical debridement). Patients were excluded if they were: younger than 70 years, declined to participate, NPUAP stage 1 and 2, with a previous severe injury or surgery to the buttocks, laboratory tests indicating low hemoglobin levels and albumin levels, high level white blood cells and C-reactive protein, X-ray indicating osteomyelitis, or existence of comorbidities for anesthesia and surgery, such as diabetes with target organ complication, venous thrombosis, cancer, Parkinson’s disease, dementia or receiving end of life care.

### Design of CKF and harvest of the flap

The anatomical basis of CKF for coverage of sacral PUs is the superior gluteal artery perforator-based fasciocutaneous/myocutaneous, bilateral symmetrical advanced (or rotated) flap of buttock.

All the operations were carried out by the same surgeon under general anesthesia. All the patients were placed in prone position for surgery. The wound was carefully and radically debrided, and the size of the resultant wound measured. The size of unilateral flap was designed 1–2 cm larger than the half-size of the wound. The perforator of the superior gluteal artery was explored by laser Doppler imaging examination preoperatively.

**Fig. 1:**
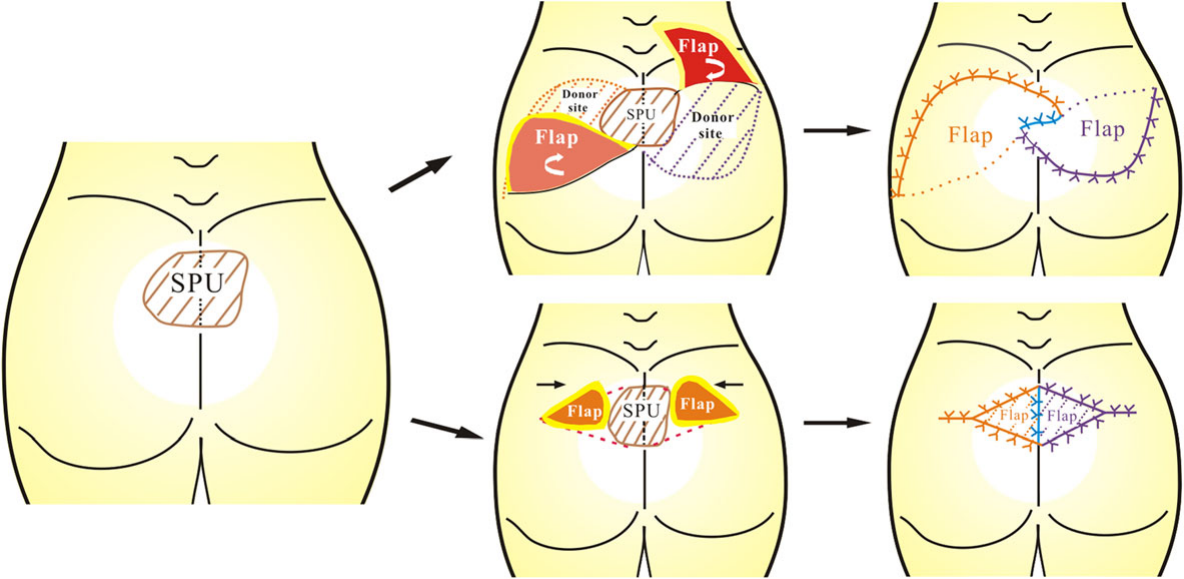
Schematic diagram of couple-kissing flap for the treatment of SPU. The flap was harvested, elevated, and rotated (or advanced) into the midline of the resultant sacral defect following debridement, and sutured primarily. The orange area and the purple area indicate one of the flaps, respectively. The blue line indicate the kissing site (interface of the two flaps). SPU: sacral pressure ulcer

Dissection was initiated from one side of buttock, the flap was elevated as a fasciocutaneous flap by dissecting the layer between the fascia and the muscle (some gluteus maximus muscle fibers were dissected depending on the remaining tissue following debridement when tissue was removed to alleviate pressure after surgery). The bilateral couple flaps were cut and then elevated and advanced (or rotated) to fit the shape of the resultant wound following debridement, respectively. A schematic diagram of the flap is shown in Fig. 1.

After successful harvest of bilateral couple flaps, the interface of the two flaps (kissing site) was sutured without any tension. The donor sites of flap were primarily closed simultaneously. Lastly, two suction drains were placed.

### Postoperative management

All patients received a standard postoperative regimen that included the suction drainage tubes were maintained for at least 1 week, stayed in intensive care unit for at least 3 days, maintenance of fluid and electrolyte balance, antibiotics were administrated intravenously based on the results of tissue culture from deep tissue after debridement performed during operation. Blood transfusion or albumin transfusion were given based on the laboratory results. Enteral nutritional support was administered when oral feeding was poor. An air suspension therapy bed (product model XFCH-01, Ningbo Yi Long Medical Equipment Co., Ltd., Zhejiang Province, China) was used by all patients postoperatively to avoid pressure on the flap and to prevent incision dehiscence, necrosis of the flap, or occurrence/recurrence of PU, without changing position of the patient. Wound dressing was changed every day.

### Data collection

Demographic information from each patient was collected, including age, sex, body mass index (BMI), location and size of defects, comorbidities, duration of PU before admission, complications, time of operation and recurrence rates.

## Results

Between July 2012 and December 2015, 17 elderly patients were admitted to our unit; five subjects were excluded, according to inclusion and exclusion criteria. A total of 12 individuals were involved in this study, including seven males and five females, with an average age of 76.83 years (ranged from 71 to 92 years). The mean BMI was 19.6 (ranged from 18.7 to 22.3). The average duration of PUs before admission was 2.9 months (ranged from 0.8 to 5 months) (Table 1).

**Table 1:** Characteristics of geriatric patients with severe sacral PUs managed with couple-kissing flap

No.	Sex	Age (years)	Predisposing factor	Duration of PUs (months)	Defect location of PUs	Sacral defect size (length × width)	Identified pathogens of the wound	Operation time (mins)	Suture removal (days)	Follow-up (months)
1	F	72	Stroke	1.5	Sacral and ischial tuberosity	7.0 × 4.5	*Pseudomonas aeruginosa*	65	15	13
2	M	74	Stroke	2.5	Sacral	6.0 × 5.0	*Enterobacter cloacae*	90	14	13
3	M	71	Spinal cord injury	5.0	Sacral and trochanteric	5.0 × 7.0	MRSA	75	16	14
4	F	82	Stroke	0.8	Sacral and ischial tuberosity	6.5 × 4.0	*Pseudomonas aeruginosa*	60	15	9
5	F	75	Stroke	3.5	Sacral	7.0 × 5.5	*Enterobacter cloacae* and *Acinetobacter baumannii*	85	15	14
6	M	92	Spinal cord injury	2.8	Sacral and trochanteric	8.0 × 10.0	*Pseudomonas aeruginosa*	90	16	12
7	M	71	Stroke	1.3	Sacral	6.0 × 4.5	Negative	105	14	24
8	F	73	Stroke	4.2	Sacral and lateral malleolar	6.0 × 7.0	*Enterobacter cloacae*	75	15	16
9	M	75	Stroke	3.3	Sacral	7.5 × 6.0	*Pseudomonas aeruginosa*	80	14	13
10	M	80	Senile valetudinariarianism	2.9	Sacral and trochanteric	5.5 × 5.0	Negative	95	16	12
11	F	82	Stroke	4.5	Sacral and trochanteric	7.0 × 6.5	*Staphylococcus aureus*	85	15	12
12	M	75	Severe malnutrition	2.6	Sacral	8.5 × 6.0	Negative	115	17	11

*F* female, *M* male, *MRSA* methicillin-resistant Staphylococcus aureus

The etiologies of sacral PUs were identical among patients confined to bed for extended period due to different reasons, including sequelae of stroke (8 cases), spinal cord injury (2 cases), senile valetudinarianism (1 case), and severe malnutrition (1 case). Of the 12 elderly patients, ischial tuberosity PUs were found in 2 cases, trochanteric PUs in 4 cases, and lateral malleolar PU in 1 case. These PUs were treated and healed by dressing change (6 cases) or closed by debridement and primary suture (ischial tuberosity, 1 case).

Results of bacterial culture of wound swab were positive in 9 cases; *Pseudomonas aeruginosa* was found in 4 cases. *Staphylococcus aureus* infection was present in 2 cases (one was methicillin-resistant *Staphylococcus aureus*). *Enterobacter cloacae* infections were present in 2 cases; coexistence of *Enterobacter cloacae* and *Acinetobacter baumannii* infection was found in 1 case*.*

The average operating time was 85 min (ranged from 60 to 115 min). All the flaps survived completely without partial or total loss. No complications related to the surgery (such as seroma, hematoma, or infection) were noted in any of the cases. The average time for removal of sutures was 15.2 days (ranged from 14 to 17 days).

All the patients were discharged to home when the suture was removed without dehiscence, with 10 patients were bedridden and 2 patient moved relied on wheelchairs.

Follow-ups by telephone or multimedia message were conducted from 9 months to 2 years (mean follow-up time was 13.6 months). The results were both functionally and aesthetically satisfactory, with no occurrence of the incision breakdown or recurrence of PU.

### Case 1

**Fig. 2:**
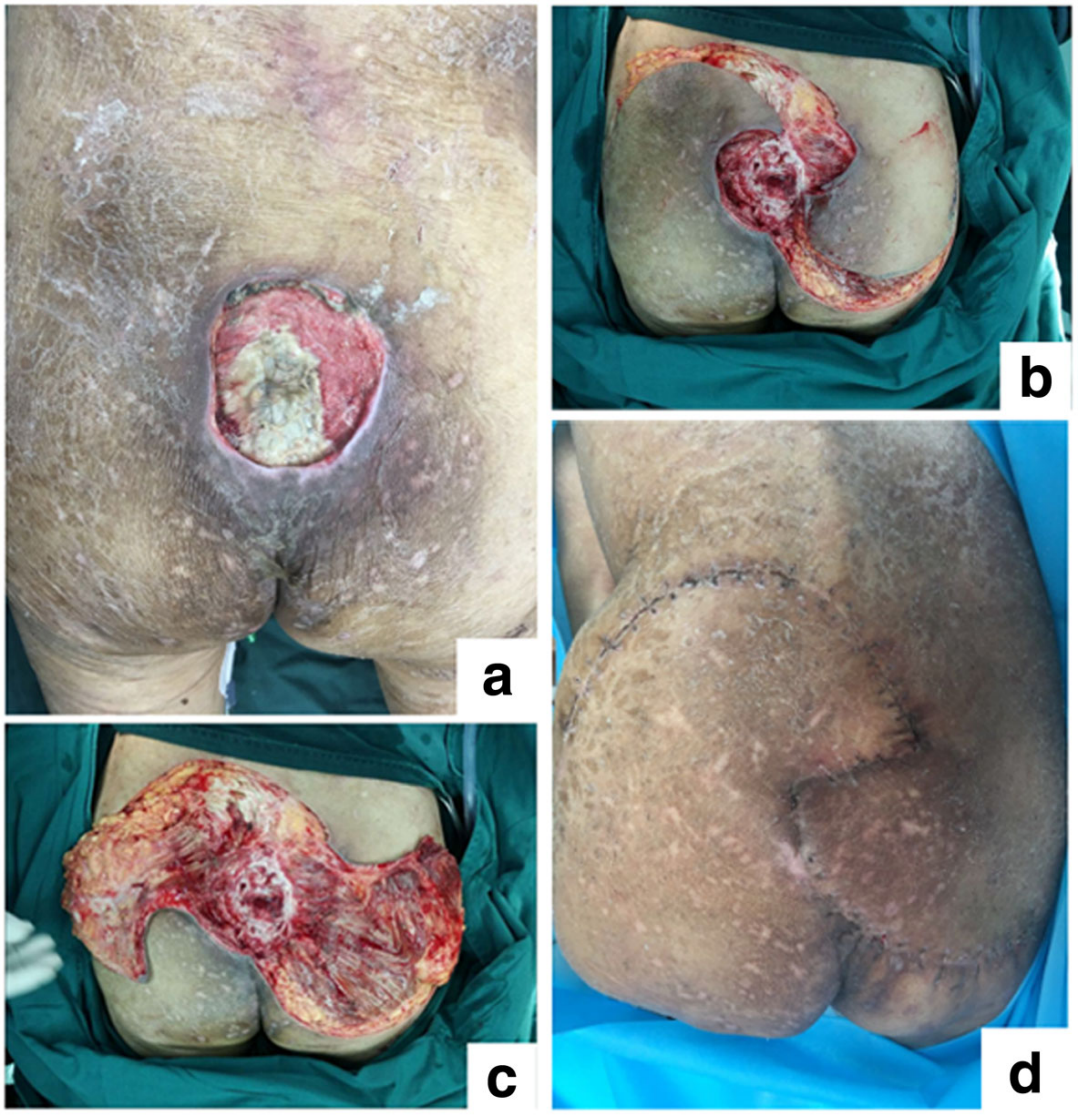
SPU in case #1 patient. **a** Preoperative view of refractory SPU covered with necrotic tissue and secretions. **b** and **c** The wound was debrided radically, resulted in a 7 cm × 9 cm full-thickness soft-tissue defect. The resultant defect was closed by rotated couple-kissing flap in the shape of *Tai-Chi*. **d** The flap survived completely, with no complication at 2 weeks postoperatively. An excellent outcome was observed

A 73-year-old woman suffering from refractory sacral PU for more than 4 months was referred to our department for reconstructive surgery (Fig. 2a). Radical debridement was performed immediately and the resultant wound was closed primarily with CKF (Fig. 2b-c). The flap was fully maintained postoperatively (Fig. 2d). After 15 months, a follow-up demonstrated satisfactory results with no recurrence.

### Case 2

**Fig. 3:**
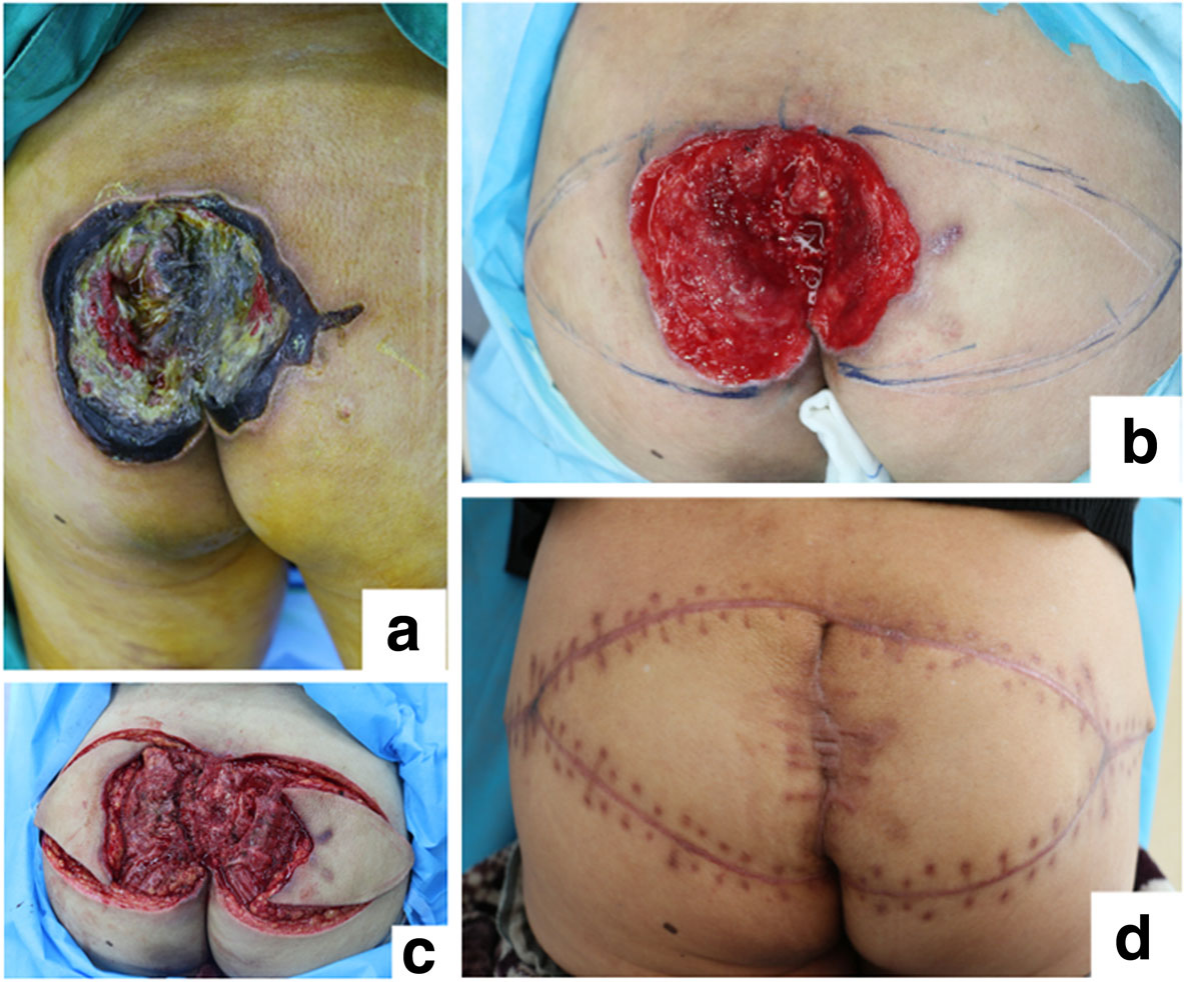
SPU in case #2 patient. **a** Severe SPU covered with necrotic tissue and secretions. **b** The wound was debrided radically, and negative pressure wound therapy was administrated to prepare the wound bed. **c** Advanced couple-kissing flap was designed and performed to close the wound after radical debridement. **d** 2 weeks after the surgery, the flap survived completely with no complications. An excellent outcome was observed

A 75-year-old man suffering from severe sacral PU for more than 3 months with necrotic tissue and secretions (Fig. 3a) was referred to our department. Radical debridement was performed immediately, and negative pressure wound therapy was administrated to prepare the wound bed until granulation tissues had formed (Fig. 3b). The wound was closed primarily with advanced CKF (Fig. 3c). 2 weeks after the surgery, the flap had survived completely with no complications. An excellent outcome was observed (Fig. 3d).

### Case 3

**Fig. 4:**
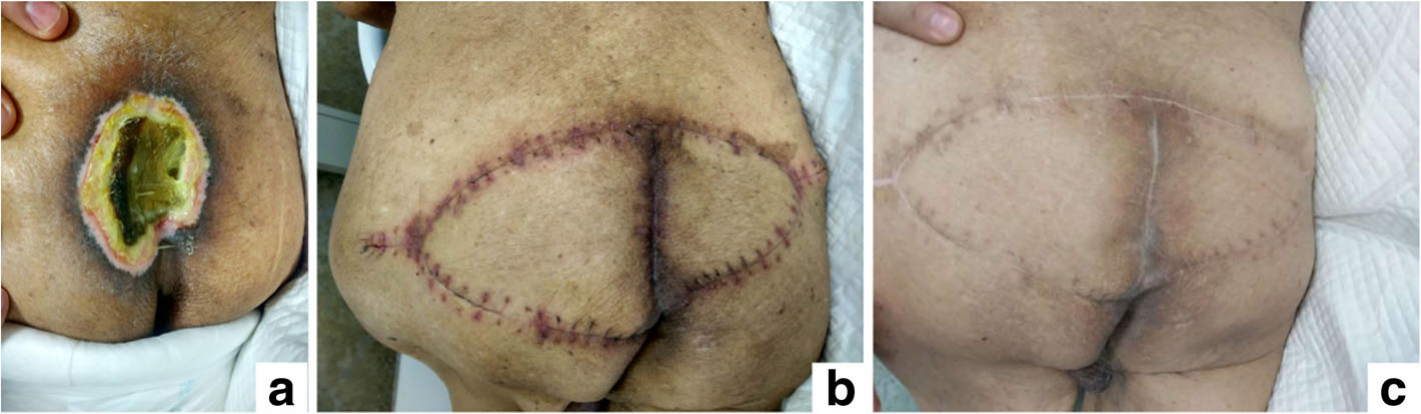
SPU in case #3 patient. **a** Preoperative view of extensive SPU with necrosis and secretions in a 92-year-old man. **b** Wound was debrided and closed with an advanced couple-kissing flap; 2 weeks following surgery the flap had remained intact. **c** Satisfactory outcome was obtained at the 2-months follow-up

A 92-year-old man with spinal cord injury accompanied with severe PU, as shown in Fig. 4a, was referred to our department. The wound was debrided to leave a defect with exposure of a tendon. The plan for sacral reconstruction was devised, using CKF and the donor site of the flap was closed primarily (Fig. 4b). At 10-month follow-up, the flap remained intact with no evidence of suture breakdown (Fig. 4c).

## Discussion

Management of severe sacral PUs remains challenging for plastic surgeons, especially for elderly patients with higher risks of anesthesia and surgery due to their particular pathophysiology characteristics and the complicated preexisting comorbidities [20]. Herein, we introduced our novel experience using CKF for the treatment of severe sacral PUs in elderly patients and obtained satisfactory outcomes both functionally and aesthetically.

In this case series, negative pressure wound therapy was mainly used for wound preparation when the wound bed was not suiss for surgery due to necrosis and/or infection or when the patient was too weak to receive the operation immediately. Although successful experience with negative pressure device in traumatic paraplegia patients with sacral PUs of stage 3 and 4 has been reported [13, 21], the ability of negative pressure wound therapy in wound infection control remains to be investigated [22, 23].

Good results have been reported using advancement island flap, split flap, and rotation flaps including gluteus maximus muscular flap and myocutaneous flap for sacral PUs reconstruction [24]. The muscle is thought to provide a better “tissue buffer” over the sacrum for continuous external tension to effectively prevent recurrence and for better ability in obliterating any dead space or healing tissue infection [25–27]. However, a recent retrospective study indicated that musculocutaneous flaps are as good as fasciocutaneous flaps in the reconstruction of PUs and there were no significant differences in early complications, postoperative morbidity, or ulcer recurrence between these two kinds of flap [28].

The superior gluteal artery perforator-based fasciocutaneous flap and gluteus maximus myocutaneous flap were both applied in this case series. Optimal outcomes were consistently obtained with no difference in the occurrence of postoperative complication or recurrence of PU.

In this case series, the fasciocutaneous flap was harvested without well-known perforators during the surgery, while reliable outcomes were also obtained and the well vascularized flap could be performed without microsurgical dissection. Similarly, Demiryilmaz et al. [29] successfully treated sacrococcygeal pilonidal sinus disease with bilateral fasciocutaneous V-Y advancement flap following total excision. Han et al. [24] closed the sacral PUs with bilateral combined V-Y fasciocutaneous advancement and gluteus maximus muscle rotational flaps. Hsiao and Chuang [30] reported their successful experience of reconstructing sacral PUs with dual-dermal-barrier fashion flaps. Prado et al. [31] treated sacral defects with “double-A” bilateral flaps based on perforators.

Partial/complete flap loss or incision dehiscence may occur following reconstructive surgery. Also, the interface of the two elevated flaps (the kissing site) may not heal primarily due to the poorer blood flow following harvesting. Moreover, the patient’s position needs to be changed every 1–2 h following sacral reconstructive flap surgery to avoid continuous external pressure, which may cause flap necrosis or incision dehiscence; patients remained prone or on their side for approximately 2 weeks until the flap was healed [17]. Fortunately, no flap loss or incision dehiscence was found in this case series.

In addition to the reliable blood supply of the flap, another possible explanation for the favorable outcome maybe our special care management. The air suspension therapy bed, which is mostly used in management of severe burn patients, was used in all patients postoperatively which could relieve external pressure on the flap and therefore prevent dehiscence of the incision, necrosis of flap or occurrence/recurrence of PU. Additionally, this method also benefited patients in that the patients could remain the supine position which was very important for the elderly patients, because this may relieve the constraint on respiration due to prone position. We recommend that this method could be part of therapeutic plan when treating severe sacral PUs, especially in elderly patients who cannot often change their position in bed.

Presence of bacterial infection is a common complication in chronic wounds. Severe and long-lasting wound infection may significantly delay the healing time, or result in the occurrence of refractory/non-healing wound [32]. Despite the results of bacterial cultures suggesting high positive infection rate of the PUs wounds (9/12, 75%) prior to surgical intervention, no sign of infection was noted postoperatively in our cases. This may be partly attributed to the fasciocutaneous flap’s ability to control postoperative infection [33]. Meanwhile, radical debridement and washout during surgery reduced the bacterial load on the wound significantly. Dressing change and administration of culture-sensitive antibiotics intravenously in the perioperative period may also account for the absence of postoperative infection.

The limited number of patients and short period in follow-up were the drawbacks of this study. Actually, practicing a regular follow-up in elder patients is not easy in this case series, since most of the patients were bedridden for long periods of time because of paraplegia or hemiplegia, therefore, nearly all the follow-ups in this study were performed by telephone or multimedia message. Due to either old age, acute or chronic diseases, some patients died and dropped out during follow-up, thus resulting in a relatively short average follow-up period in this case series. However, the legal guardians of these patients reported satisfactory outcomes and no recurrence or breakdown of the incisions were reported.

Although many therapeutic modalities have been suggested for the treatment of PUs [13–17], however, recurrence remains one of main challenges in the treatment of PUs. Study of Sameem et al. [34] indicates that, recurrence rate of musculocutaneous flaps, fasciocutaneous flaps and perforator-based flaps for treatment of PUs were 8.9%, 11.2% and 5.6%, respectively. At the mean 13.6-month follow-up in this series, no recurrences were detected. In our study, all the surgeries were performed by the same experienced surgeon in a single institution. Meanwhile, nursing care, especially regular changing of the position of the patients every 1–2 h, has been stressed to all the patients and guardians and was provided at home to prevent the occurrence/recurrence of PU. Furthermore, follow-up by telephone or multimedia message in this study has the advantage of timely communication whenever sign of PU appeared.

The CKF procedure provides the following advantages for coverage of PUs: (1) reliable flap success rate. Because of the resultant wound following debridement was covered by couple flaps, the size of each flap needs not to be too large, so the damage to the donor site of the flap is relatively small and can be usually closed primarily; (2) the damage of this approach is relatively small which can be performed safely even in elderly patients; (3) the symmetrical appearance of CKF was aesthetically satisfactory; and (4) the recurrence of the CKF approach is lower.

## Conclusions

Management of severe sacral PUs among elderly patients remains challenging for surgeons because of complications and unexpected sequelae. The CKF approach is simple to implement, while providing excellent coverage of severe sacral PUs, even in frail elderly patients. The recurrence rate associated with this method is very low if appropriate postoperative nursing care is provided. Indeed, the CKF approach can be a valuable choice for surgeons and should be highly recommended in the reconstruction of sacral PUs.
